# Enhancing Functional Properties and Shelf Life of *Oryza sativa* L. via Grain Stabilization Techniques

**DOI:** 10.3390/foods14040596

**Published:** 2025-02-11

**Authors:** Eunsu Song, Hyeeun Gwon, Jaeyoung Choi, Md Atiqual Islam, Jiyeon Chun, Yun Hee Chang, Jinah Hwang

**Affiliations:** 1 Department of Food and Nutrition, College of Natural Sciences, Myongji University, Yongin 17058, Republic of Korea; eunsu4979@gmail.com (E.S.); yhchang@mju.ac.kr (Y.H.C.); 2 Department of Nutrition and Food Engineering, Daffodil International University, Dhaka 1216, Bangladesh; atiquali@gmail.com; 3 Department of Food Science and Technology, Sunchon National University, Suncheon 57922, Republic of Korea; cjyfall@scnu.ac.kr; 4 Bio-Healthcare Food Research & Analysis Center, Sunchon National University, Suncheon 57922, Republic of Korea

**Keywords:** *Oryza sativa*, resistant starch, bioactive compounds, storage stability, post-harvest processing

## Abstract

**Background:** *Oryza sativa* L. is one of the main staple crops in the South Korea. While rice production has remained stable over the past decades, rice consumption has gradually declined, leading to a rapid stockpile in rice inventory. Conventional rice storage methods often fail to preserve functional properties and long-term stability, necessitating innovative processing techniques. **Methods:** To address this issue, we developed a grain stabilization technique (GST) to enhance the functionality and shelf life of white rice (WR), brown rice (BR), and rice germ (RG). The GST process was conducted in a single-batch system, integrating a controlled temperature cycle (65~85 °C) with 60 rpm rotation, far-infrared (26,400 W/m^2^), and ultraviolet (254 nm, 60,880 J/m^2^) irradiation in an enclosed chamber equipped with an exhaust system for moisture, odor, and impurity removal. The process was followed by air drying (25 °C, 15 h) to ensure stability. **Results:** The GST significantly increased resistant starch content in BR and WR by 214% and 27%, respectively, but not in RG. Additionally, GST enhanced the contents of campesterol, stigmasterol, β-sitosterol, and octacosanol in BR and RG, but not significantly in WR. Furthermore, this technique markedly reduced moisture content, acidity, and bacterial counts over a 90-day storage period and kept mycotoxin levels within safe limits in WR, BR, and RG. GST also altered the microstructures of WR and BR, indicating gelatinization and amorphization of starch granules. **Conclusions:** These findings contribute to advancing food science by presenting GST as a transformative method to extend shelf life and improve the nutritional profile of rice, aligning with global efforts to reduce food waste, improve dietary health, and develop sustainable food processing technologies.

## 1. Introduction

*Oryza sativa* L. (rice) is a vital staple food in Korea and Asia, but its consumption has steadily declined since the 1980s due to changes in dietary habits [1]. Despite this decline, rice production has surged due to high-yield cultivars and technological advancements, resulting in a large rice stockpile. This surplus necessitates innovative and sustainable rice processing methods to boost consumption and extend shelf life.

Rice quality peaks after harvest but can degrade during post-harvest processes like drying, milling, storage, and processing [2,3]. Since the harvested rice contains 22~24% moisture for medium-grain rice, proper drying is crucial to maintaining quality and preventing deterioration under different storage conditions. Milling removes the husk and bran layers, producing white rice (WR), brown rice (BR), and rice germ (RG). While BR and RG are nutritionally superior to WR, they are more prone to lipid oxidation and microbial spoilage, limiting their storage stability and consumer acceptance.

BR is whole-grain rice, consisting of pericarp, seed coat, and aleurone layers after the removal of the rice bran layer. WR undergoes further milling, which removes the bran and most of the RG, resulting in less fiber and fewer nutrients but a longer shelf life. BR is valued for its low glycemic index and high fiber content but has a coarser texture and higher lipid peroxidation susceptibility, reducing its palatability and stability [4,5]. RG is rich in macronutrients and micronutrients, including phytosterols and phytochemicals, but it is removed during rice milling to improve storage quality, despite being supplemented in cooking due to its high nutrient content. Therefore, new processing techniques are needed to enhance the storage stability and functional properties of BR and RG while maintaining consumer acceptability.

Numerous studies have attempted to develop processing methods to address the drawbacks of rice, particularly brown rice (BR). For example, Kim et al. [6] selectively removed the pericarp layer to improve the texture and physicochemical properties of BR. However, existing methods such as parboiling and selective milling often compromise nutritional value or incur high processing costs.

The Grain Stabilization Technique (GST) integrates controlled temperature cycling (65~85 °C), far-infrared, and ultraviolet irradiation (UV) to enhance functional properties such as resistant starch and phytosterol retention while significantly extending shelf life [7]. This single-batch system minimizes contamination risks and improves operational efficiency by eliminating the need for separate processing steps. Ding et al. reported that infrared radiation enhances rice texture by modifying protein structure and inhibiting oxidation, thereby improving physical quality [8]. Similarly, Ratseewo et al. confirmed that far-infrared radiation improves starch digestibility and bioactive compound retention, enhancing rice’s nutritional benefits [9]. UV irradiation further enhances microbial safety by reducing surface contamination and extending shelf life. In addition to its functional benefits, GST is energy-efficient and cost-effective, as it consolidates multiple treatments into a single system. This reduces energy consumption and processing time, making GST highly feasible for industrial-scale applications.

Starch is categorized into rapidly digestible, slowly digestible, and resistant starch (RS) based on its digestibility [7]. RS is not degraded by amylases in the body but can be fermented by microbiota in the large intestine [10]. Recently, RS has been reported to function similarly to water-soluble dietary fiber due to its low digestibility and absorption, producing beneficial metabolites for health. Accumulating evidence suggests that RS can modulate insulin sensitivity, regulate blood glucose levels [11], reduce fat accumulation, and improve lipid metabolism [12].

In this study, we investigated the effects of GST processing on the physicochemical properties, RS content, phytosterol levels, and storage characteristics of WR, BR, and RG during long-term storage. The findings provide insights into healthier rice processing alternatives and strategies for promoting rice consumption through enhanced functionality and extended shelf life.

## 2. Material and Methods

### 2.1. Sample Preparation and Storage Condition

**Figure 1:**
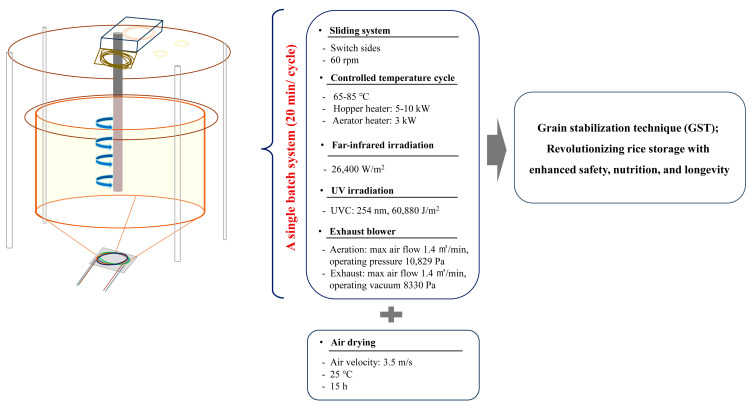
Schematic representation of the Grain Stabilization Technique (GST) for *Oryza sativa* L., illustrating key processing steps and their impact on rice storage. The process involves a controlled temperature cycle (65~85 °C), far-infrared irradiation (26,400 W/m^2^), UV irradiation (254 nm, 60,880 J/m^2^), and air drying (3.5 m/s, 25 °C, 15 h), all performed within a single-batch system (60 rpm, 20 min/cycle). These treatments collectively contribute to reducing moisture gain, acidity, and bacterial contamination, while enhancing resistant starch content, bioactive compounds, and overall shelf stability.

WR, BR, and RG of the Samgwang variety was provided by the Korea federation of Rice Bran (Seoul, Republic of Korea). The rice samples underwent a GST process in a single-step batch system, which involved a controlled temperature cycle ranging from 65 °C to 85 °C with 60 rpm (rotations per minute) in an enclosed rotating chamber (Figure 1). The rotation ensured uniform mixing and consistent temperature distribution. The controlled temperature system maintained a sequential adjustment between 65 °C and 85 °C, which is optimal for parboiling rice grains, enhancing their stability and functional properties. The process also included far-infrared irradiation (26,400 W/m^2^) for drying, sterilization, and improvement of cooking and sensory properties, and UV (254 nm, 60,880 J/m^2^) for microbial sterilization, extending the shelf life and improving the safety of the grains. Impurities such as moisture, odors, grain husks, and fine debris were removed during this process.

The entire GST process was completed in a single 20-min cycle, followed by air drying at ambient temperature (25 °C) for 15 h using a controlled airflow system with an air velocity of 3.5 m/s to remove any remaining heat, moisture, or odors. The samples were stored as intact grains under controlled conditions. Controlled storage conditions were set at 25 °C and 74% humidity, reflecting the average summer conditions (June to August) in 10 major Korean cities from 2015 to 2017 [1]. Samples were collected every 30 days for 90 days. For experimental analyses, the stored samples were preserved at −80 °C to ensure stability, then initially ground using a Vitamix pro 780 blender (Vitamix, Cleveland, OH, USA) and subsequently passed through a 100-mesh sieve to achieve uniform particle size before further experiments.

### 2.2. Moisture Content Analysis

The moisture content of the rice was analyzed using the official method of analysis [13]. In brief, 5 g of powdered sample was weighed and dried in an oven (Model 0610, Dongyang Science, Gwangju, Republic of Korea) at 105 °C for 3 h, then reweighed after cooling in a desiccator. Moisture content was calculated based on the weight loss.

### 2.3. Resistant Starch

The resistant starch (RS) content was determined using the official method 2002.02 [14] with a Resistant Starch Assay Kit (Megazyme, Wicklow, Ireland) according to the manufacturer’s instructions. Briefly, 0.1 g of rice sample was mixed with 4 mL of a pancreatin and amyloglucosidase solution, and the mixture was incubated in a water bath (Maxturdy-18, Daihan Scientific Co., Wonju, Republic of Korea) at 37 °C with 200 rpm for 16 h. After centrifugation at 2914× *g* for 10 min using a swing-bucket rotor with a radius of 165.3 mm (VS-550, Vision Scientific Co., Bucheon, Republic of Korea), the supernatant was decanted, and 8 mL of 50% ethanol was added to the pellet, a process that was repeated twice. Then, 2 mL of 2 M KOH was added to the final pellet, and the mixture was stirred in an ice bath. The mixture was then incubated in a shaking water bath at 50 °C for 30 min after adding 8 mL of 1.2 M sodium acetate buffer (pH 3.8) and 0.1 mL of amyloglucosidase (3300 U/mL). Finally, after centrifugation at 2914× *g* (VS-550) for 10 min, 0.1 mL of the supernatant was mixed with 3 mL of glucose oxidase/peroxidase reagent, incubated at 50 °C for 20 min, and the absorbance was read at 510 nm to measure hydrolyzed glucose content. The RS content was calculated using hydrolyzed glucose as an internal standard and expressed as RS (g/100 g DW).

### 2.4. Phytosterol and Policosanol Analysis

The lipophilic components, phytosterols and policosanol, in rice were quantitatively analyzed following the method described by Kim et al. [6]. The contents of phytosterols and policosanol were quantified using gas chromatography analysis with a flame ionized detector (GC-FID). Separation of phytosterols was performed using an HP 5890 Series II (Hewlett-Packard Co., Palo Alto, CA, USA) GC system equipped with an HP-5 capillary column (30 m × 0.32 mm × 0.25 μm, Agilent Technologies, Santa Clara, CA, USA) and a flame ionizing detector (FID, H2: 30 mL/min and air: 300 mL/min; Agilent Technologies). The injection volume was 1 μL, and the flow rate of the carrier gas, nitrogen, was 3 mL/min. The injection and detection temperatures were 320 °C and 300 °C, respectively. Phytosterols were separated on a column set at 250 °C for 15 min and then elevated to 300 °C at a rate of 15 °C/min. Quantitative analysis of phytosterols and policosanols was performed using the peak area and relative response factor of a 0.1% internal standard (5α-cholestane; Sigma-Aldrich, St. Louis, MO, USA), injected at concentrations of 1:2, 1:1, and 2:1 (*v*/*v*). Results were expressed as mg/100 g dry weight (DW).

### 2.5. Acidity Analysis

The oil from rice samples was extracted using the Folch method [15], and acidity was measured with an acidity analyzer (CDR Foodlab Junior, Florence, Italy). Briefly, 3 g of rice powder was mixed with 30 mL of a chloroform–methanol solution (2:1, *v*/*v*) and extracted at 4 °C with 200 rpm for 3 h in a multi-spin shaker (KMC-8480MX4DT-C, Vision Scientific Co.). After extraction, the oil sample was filtered and the solvent was evaporated (Eyela A-1000S, Tokyo, Japan). Acidity was expressed as a percentage of oleic acid.

### 2.6. Bacteria Counting

Bacterial counting was performed using the official method of analysis [13]. Briefly, 1 g of sample was homogenized with 9 mL of distilled water. Serially diluted samples were then added onto petrifilm aerobic count plates (3M Petrifilm, 3M, Saint Paul, MN, USA) and incubated at 35 °C for 36 h. The colonies were counted using a colony counter and expressed as colony-forming units (CFU) per gram.

### 2.7. Aflatoxin Isolation and Analysis

The isolation and analysis of total aflatoxin, deoxynivalenol, zearalenone, and ochratoxin A were performed following the aflatoxin analysis method outlined in the Food Code by the Ministry of Food and Drug Safety [16]. To isolate aflatoxins from rice powder and to confirm changes in aflatoxin levels after the grain stabilization process, these mycotoxins were analyzed using LC-MS/MS (Agilent Technologies, Santa Clara, CA, USA) with a C18 column (4.6 × 250 mm, 5 μm), in accordance with the Food Code [16].

### 2.8. Scanning Electron Microscopy

Changes in the microstructure after the grain stabilization process were observed using scanning electron microscopy (SEM, Hitachi SU-70, Tokyo, Japan). The rice samples were cut to approximately 100 μm thickness and coated with gold for 90 s using a sputter coater to enhance conductivity and prevent charging. Observations were carried out in a high-vacuum chamber at an acceleration voltage of 15 kV, with magnifications ranging from 25× to 3000×.

### 2.9. Statistical Analysis

All experiments were performed at least in triplicate, and the results were expressed as mean ± standard error of the mean (SEM). Statistical analysis was conducted using SPSS (Version 23, IBM-SPSS, Armonk, NY, USA), with significance set at *p* < 0.05. To determine significant differences among samples across storage periods, a one-way ANOVA was performed, followed by Duncan’s multiple range test for post-hoc comparisons. Significant differences between samples with and without GST treatment were analyzed using an independent sample *t*-test.

## 3. Results

### 3.1. Moisture Content

The initial moisture content (Day-1) is shown in Table 1, and changes were monitored over time to the final day (Day-90). Initially, WR had the highest moisture content among the samples, followed by BR and RG. Over time, the moisture content generally increased across all rice types. Notably, after GST processing, BR showed a more controlled increase in moisture content, with a rise of 6.2% over the 90 days compared to a 12.4% increase in non-processed BR.

For RG, a different trend was observed. Post-processing, RG samples displayed a smaller increase in moisture content (16.2%) compared to the non-processed RG (20.6%) over the same period. However, despite this general trend, the GST-processed RG still maintained a significantly lower moisture content after 90 days than the non-GST-processed RG.

In all types of rice, the GST process effectively suppressed the increase in moisture content when compared to non-processed samples over the 30, 60, and 90-day storage periods, with these differences being statistically significant.

### 3.2. Resistant Starch Analysis

The RS levels in the rice samples were measured both before and after GST processing. In the non-processed rice samples, the RS levels were 1.38 g/100 g DW for BR, 1.89 g/100 g DW for WR, and 0.20 g/100 g DW for RG. After GST processing, the RS levels increased to 4.33 g/100 g DW for BR-GST, 2.40 g/100 g DW for WR-GST, and 0.25 g/100 g DW for RG-GST (Table 2).

The RS content in BR increased significantly after GST processing, showing a remarkable 214% increase. In WR, the RS content increased by 27%, while in RG, there was a 25% increase. These findings suggest that the GST process is particularly effective in converting digestible starch into resistant starch in BR, though less pronounced effects in WR and RG.

### 3.3. Phytosterol and Policosanol in Rice

The GST process significantly influenced the levels of phytosterols (campesterol, stigmasterol, and β-sitosterol) and octacosanol in WR, BR, and RG. After GST processing, both BR and RG showed substantial increases in phytosterol content, while WR exhibited minimal changes (Table 3). The most notable reduction was observed in BR, where campesterol levels decreased by approximately 40%.

Among all rice samples, RG and RG-GST contained the highest levels of phytosterols and octacosanol. In contrast, the phytosterol and octacosanol contents tended to decrease in WR and WR-GST after processing. Over the 90-day storage period, the phytosterol content of untreated BR and RG showed a significant decline, while BR-GST and RG-GST exhibited a more gradual decrease. Specifically, the decrease in phytosterol content ranged from 11~13% in BR-GST and 5~10% in RG-GST, whereas the non-processed BR experienced the most pronounced reduction, with phytosterol levels dropping by 11% to 37%, followed by WR, which showed reductions ranging from 3% to 15%. Interestingly, RG-GST not only showed slight improvements in phytosterol content but also maintained its octacosanol levels better than non-processed RG during the storage period.

### 3.4. Free Fatty Acid Value

**Figure 2:**
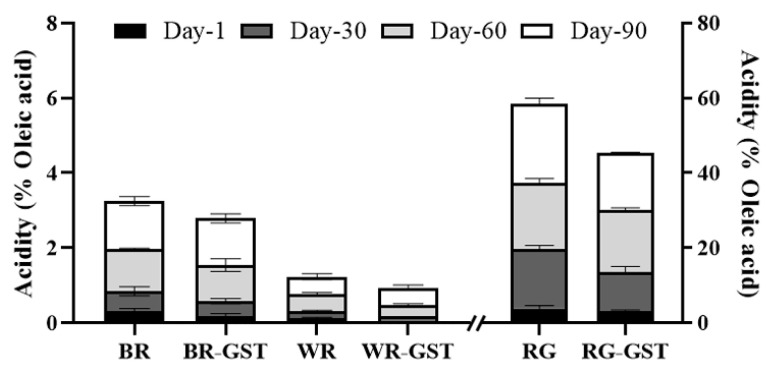
Changes in acidity (% oleic acid) over time in non-processed and GST-processed rice groups over a 90-day storage period. WR: white rice; BR: brown rice; RG: rice germ; GST: grain stabilization technique. Data are presented for Day-1, Day-30, Day-60, and Day-90.

The FAV, expressed as acidity (% oleic acid), in rice samples increased over the storage period, with the highest values observed in RG (Figure 2). The GST processing effectively reduced the FAV in both BR and WR, with significant differences observed from 60 days of storage onwards, indicating that GST processing helps to mitigate the increase in free fatty acids. For RG, although the FAV increased over time, the RG-GST samples showed a noticeably lower FAV compared to non-processed RG at 90 days, suggesting that GST can reduce oxidation and improve the storage stability of rice.

### 3.5. Bacterial Counting

The bacterial counts in rice samples decreased significantly after GST processing (Table 4). All post-processed rice (BR-GST, WR-GST, RG-GST) showed significantly lower bacterial counts compared to their non-processed counterparts, with the greatest reductions observed in WR, followed by RG and BR. On the first day of storage, the bacterial counts in post-processed rice were reduced by 7.0% in BR-GST, 24.2% in WR-GST, and 13.8% in RG-GST compared to the respective non-processed samples. By day 90, the bacterial counts further decreased, with BR-GST showing a reduction to 4.71 log CFU/g DW, WR-GST to 1.37 log CFU/g DW, and RG-GST to 3.56 log CFU/g DW. These reductions correspond to decreases of 4.5%, 18.2%, and 21.9%, respectively, from the initial bacterial counts. These data suggest that the GST process is effective in reducing bacterial contamination and enhancing the microbial safety of rice during storage.

### 3.6. Aflatoxin Analysis

The levels of mycotoxins, including total aflatoxins (sum of M1, M2, G1, and G2), deoxynivalenol (DON), zearalenone (ZEA), and ochratoxin A (OTA), were measured in pre- and post-processed rice samples as shown in Table 4. No total aflatoxins or DON were detected in any of the rice samples. However, ZEA and OTA were detected in the RG group. Specifically, non-processed RG contained 9.61 μg/kg DW of ZEA and 0.91 μg/kg DW of OTA. After GST processing, the RG-GST samples showed 17.01 μg/kg DW of ZEA and 0.78 μg/kg DW of OTA after processing. Although ZEA levels increased following GST processing, both ZEA and OTA levels in RG-GST remained well within the acceptable limits set by Korean food safety guidelines [16], which are 100 μg/kg DW of ZEA and 5 μg/kg DW of OTA.

This indicates that while GST processing may not significantly reduce the levels of ZEA and OTA in RG, it effectively keeps these levels within safe limits, ensuring the rice’s safety for consumption.

### 3.7. Microstructure of Rice

**Figure 3:**
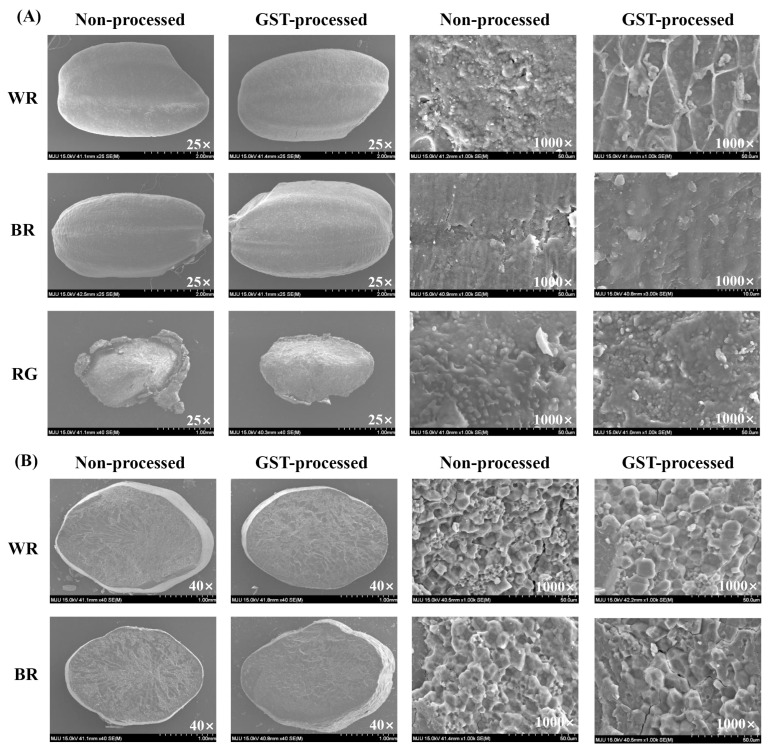
Scanning Electron Microscopy (SEM) images comparing the external surfaces and cross-sections of non-processed and GST-processed rice samples. (**A**) External surface images of white rice (WR), brown rice (BR), and rice germ (RG) at 25× and 1000× magnifications. (**B**) Cross-section images of WR and BR at 40× and 1000× magnifications. The images highlight the microstructural changes induced by the GST process, showing differences in surface texture and internal structure between non-processed and GST-processed samples. GST: grain stabilization technique.

SEM revealed significant changes in the microstructure of rice samples after GST processing (Figure 3). At 25× magnification, the external surfaces of BR and WR showed no noticeable differences before and after processing. However, the RG sample exhibited trimmed edges, likely due to continuous collisions during the GST process.

At a higher magnification of 1000×, the external surfaces of BR-GST and WR-GST displayed signs of protein agglomeration and starch gelatinization, effects of the stabilization process. The cross-sectional images at this magnification further highlighted these changes; the starch granules in the non-processed BR and WR were polygonal and polyhedral, whereas in BR-GST and WR-GST, these granules appeared dulled and fused, suggesting amorphization and gelatinization induced by the GST process. For RG-GST, the SEM images showed trimmed edges and a significantly altered microstructure compared to the non-processed RG, consistent with the mechanical and thermal effects of the stabilization technique.

## 4. Discussion

This study evaluated the effects of GST on the functional properties and shelf life of WR, BR, and RG. The findings demonstrate that GST significantly enhances rice stability by reducing MC, increasing RS content, preserving bioactive components, reducing bacterial contamination, and maintaining safe levels of mycotoxins in rice.

MC is a crucial factor in rice deterioration, affecting microbial growth and shelf stability [17]. At harvest, rice contains ~20% moisture, which decreases to ~15% during threshing [18]. The ideal MC for long-term storage and prevention against deterioration is 12–14% at 25 °C [19,20]. In this study, GST effectively maintained MC within safe limits, particularly in BR and WR, preventing excess moisture gain and ensuring storage stability. This aligns with findings by Genkawa et al. [17] and Makky et al. [19], which emphasize that proper moisture control is crucial for long-term rice storage.

RG, in contrast, typically has a lower moisture content than whole rice grains due to its unique composition. A recent study reported that RG contains lower MC (10.53 ± 0.30 g/100 g) compared to BR and WR [21]. This lower moisture level reduces microbial contamination risks but increases susceptibility to oxidative rancidity due to its high lipid content. Thus, proper stabilization, such as GST, is essential to preserving its quality. Infrared treatment further contributes to MC reduction, positively impacting rice shelf life [22]. Additionally, according to Palacios-Cabrera [23], rice stored with 13% MC at 25 °C is safe at 17% humidity and with 14% humidity for 3 to 7 months. The ability of the GST process to reduce and stabilize MC across rice types, including RG, enhances its potential for long-term storage under varying environmental conditions.

RS is a non-digestible starch fraction that resists enzymatic degradation in the small intestine and undergoes fermentation in the colon, providing dietary fiber-like health benefits [22]. Its formation is influenced by starch type, granule structure, amylose–amylopectin ratio, starch crystallinity, and thermal processing [24]. Among RS types, RS1 represents physically inaccessible starch in partly polished grains or seeds; RS2 is found in raw potatoes, green bananas, and high-amylose maize starch with a tightly packed crystalline structure; and RS3 is retrograded starch formed through amylose recrystallization during moist heat treatments [25]. RS4 is chemically modified starch and RS5 forms through amylose–lipid interactions [26]. The increase in RS content in BR and WR after GST processing highlights the effectiveness of the GST process in converting digestible starch into resistant starch, particularly RS3. This conversion is beneficial for health, as RS acts like dietary fiber, producing beneficial metabolites and improving insulin sensitivity [11,12].

According to Huang et al. [26], RS content increases through amylopectin retrogradation. The higher amylose content in BR and WR compared to RG contributes to this increase, as RS3 forms under higher amylose conditions [27]. The increased rate of amylose content in BR, WR, and RG parallels the increase in RS in rice, showing an identical tendency. Infrared radiation, as a key component of the GST process, plays a significant role in increasing RS. It is recognized as an advanced heat-treatment method due to its high heat transfer efficiency and ability to directly penetrate the product without heating the surrounding air [28]. This process induces structural changes in starch and enhances starch–protein adhesion, limiting enzymatic access to starch granules and thereby increasing RS content [28]. While vacuum storage effectively inhibits microbial growth by limiting oxygen, it does not actively modify starch structure or promote RS formation. This underscores the unique advantage of GST in simultaneously improving storage stability and nutritional properties.

The RG-GST, however, showed a significantly lower increase in RS content compared to BR and WR due to its lower amylose content. Amylose is critical for the formation of RS3 and RS5, which are less likely to form in RG compared to BR and WR. Unlike BR and WR, RG is primarily composed of lipids, proteins, vitamins, and minerals, with a relatively lower starch content, including amylose. This unique nutritional composition of RG limits its capacity for starch retrogradation, a key process for RS3 formation.

Phytosterols are plant sterols, including stanols that humans cannot synthesize [29]. Major phytosterols in plants include sitosterol, campesterol, and stigmasterol, with rice bran being a significant source, containing about 22% of rice bran oil [29]. Octacosanol, a fatty alcohol, is another important bioactive compound in rice. Both phytosterols and octacosanol play roles in regulating cholesterol and lipid metabolism [29,30]. The decrease in phytosterol content in WR and RG after GST is consistent with previous studies indicating a reduction with increased milling and prolonged storage [29,31]. However, BR-GST retained higher phytosterol levels than BR, suggesting that GST may aid in the preservation of these bioactive compounds in BR.

FAV is a key indicator of rice deterioration during storage due to more rapid oxidation of fat compared to protein or starch [32]. Oxidation of fatty acids deteriorates the taste of rice and is an important quality indicator [33]. FAV is influenced by oxygen, temperature, and moisture content [34]. The lower FAV in BR and WR after GST process suggests a reduction in oxidative rancidity. [34,35]. Moreover, the increase in FAV over the storage duration may influence the decrease in phytosterol and octacosanol contents [36]. Heat and infrared treatments can halt the increase in FAV by inactivating lipase and reducing moisture content [35,37].

Common bacteria on grain are generally non-pathogenic; however, enteric pathogens such as *Salmonella* and *Escherichia coli* can contaminate through poor hygiene during transportation and storage [38]. Bacterial growth is affected by moisture content, temperature, and gas composition, with moisture content causing increases in bacteria, yeast, and molds [39]. The significant reduction in bacterial counts in GST-processed rice indicates that GST effectively reduces bacterial contamination, enhancing rice safety and shelf life. This finding aligns with previous studies on the effects of moisture control on bacterial growth [38,39]. Consequently, the decrease in bacterial count in rice samples implies a loss of moisture content during storage, as reported in previous studies.

Approximately 25~40% of consumed cereals worldwide are contaminated by mycotoxins, causing significant economic damage [40]. Aflatoxin contamination, primarily caused by *Aspergillus flavus* and *Aspergillus parasiticus*, produces hepatotoxic and genotoxic aflatoxin B1 [41]. *Fusarium graminearum* generates DON and ZEA, which are associated with various toxic effects [42]. OTA, found in rice, can cause kidney, neuro-, myelo-, and immune-toxicity [40]. In this study, the analysis of mycotoxins (total aflatoxin, DON, ZEA, and OTA) was conducted following guidelines established in the Standard and Regulation of Korean Food [16] and MR27 Milled Rice and Fortitude Milled Rice [43]. These mycotoxins are critical indicators of grain safety under storage conditions.

The presence of ZEA and OTA in RG and RG-GST within safe limits suggests that while GST does not significantly reduce aflatoxin levels, it effectively maintains these mycotoxins at manageable levels. The detected mycotoxin levels were below 18% of the action level, remaining within Korean MFDS guidelines [16], with no USDA guidelines for OTA and ZEA. These results demonstrate that the GST process effectively maintains mycotoxin levels within safe regulatory limits, even under summer-like storage conditions characterized by high temperature and humidity. This highlights GST’s potential to suppress fungal contamination and ensure microbial safety during rice storage.

SEM analysis revealed significant changes in the microstructure of rice samples after GST processing. The surface of rice was smooth, and rice starch had polyhedral granules in the blastomere and blastocyst [44]. The observed gelatinization and agglomeration of protein suggest that GST alters the physical properties of rice, potentially improving its cooking quality and taste. These changes are consistent with findings by Sittipod and Shi [45] and Ding et al. [8] on the impact of thermal processing on rice structure. During GST, rice was treated with temperatures between 65 and 85 °C, leading to amorphization and gelatinization of starch granules [46]. Hot-vapor and drying steps lead to gelatinization of endosperm starch, disrupting hydrogen bonds in amylose and amylopectin [26]. Far-infrared treatment also alters starches and proteins, inhibiting enzyme activity and affecting microstructure [8].

The GST process demonstrates scalability for industrial applications by combining cost-effective ultraviolet and far-infrared treatments to inhibit bacterial growth, reduce moisture content, and promote starch gelatinization. Unlike traditional methods that focus solely on either shelf life or nutrient preservation, GST achieves both. For instance, the significant increase in resistant starch content across all rice varieties post-GST processing highlights its potential to cater to health-conscious consumers while addressing the rapid spoilage of rice germ under normal storage conditions. Furthermore, the integration of controlled temperature cycling within the GST process enables effective parboiling, which enhances grain stability and functionality without additional handling. This study also confirms that GST reduces microbial contamination while maintaining bioactive compounds, aligning with the goal of producing safer, nutritionally enriched grains for extended storage periods.

In comparison to traditional drying methods, such as sun drying or hot air drying, GST achieves more uniform moisture reduction while requiring less processing time due to the penetrating effect of far-infrared radiation [8]. Similarly, unlike vacuum storage, which prevents microbial growth by limiting oxygen but does not actively reduce moisture, GST simultaneously stabilizes moisture levels and inhibits microbial contamination. These functionalities make GST a superior approach for long-term storage.

## 5. Conclusions

Rice is a pivotal source of nutrition, providing 50% of the human diet due to its ease of cultivation, affordability, and longer shelf life compared to other carbohydrate-rich foods. This study demonstrates that the GST, integrating controlled temperature cycling (65 °C to 85 °C), far-infrared, and UV in a single-batch process, and air drying, can significantly enhance the shelf life and functional properties of rice. The retention of bioactive components, increased RS content, and reduced microbial contamination underscore GST’s effectiveness in improving both the nutritional quality and storage stability of rice.

The GST process consolidates multiple steps into a single, scalable system, reducing contamination risks and operational complexity while promoting energy efficiency. These advancements position GST as a transformative solution for mitigating food waste and enhancing global food security. Its potential for broader application across various grains further emphasizes its value as a modern food processing innovation.

## Figures and Tables

**Table 1:** Moisture content (%) in non-processed and GST-processed rice groups over a 90-day storage period.

Moisture Content (%)						
**Storage**	**BR**	**BR-GST**	WR	WR-GST	RG	RG-GST
Day-1	11.3 ± 0.46 ^a^	11.3 ± 0.06 ^a^	14.4 ± 0.04 ^b^	13.4 ± 0.60 **	8.69 ± 0.49 ^a^	7.14 ± 0.33 ***
Day-30	11.6 ± 0.26 ^ab^	11.5 ± 0.25 ^a^	13.7 ± 0.35 ^a^	13.2 ± 0.47	9.04 ± 0.23 ^a^	7.88 ± 0.35 ^b^***
Day-60	11.8 ± 0.12 ^b^	11.6 ± 0.08 ^a^*	13.9 ± 0.11 ^a^	13.0 ± 0.09 ***	9.63 ± 0.14 ^b^	8.70 ± 0.22 ^c^***
Day-90	12.7 ± 0.39 ^c^	12.0 ± 0.15 ^b^**	14.0 ± 0.35 ^a^	13.3 ± 0.26 **	10.10 ± 0.19 ^c^	8.61 ± 0.830 ^c^**

WR: white rice; BR: brown rice; RG: rice germ; GST: grain stabilization technique. Data are presented as means ± SEM (*n* = 3). Different letters (a–c) indicate significant differences (*p* < 0.05) within non-processed and GST-processed groups. Significant differences between non-processed and GST-processed samples are indicated by independent *t*-test (* *p* < 0.05; ** *p* < 0.01; *** *p* < 0.001).

**Table 2:** Resistant starch (g/100 g) in non-processed and GST-processed rice groups.

Resistant Starch (g/100 g)					
BR	BR-GST	WR	WR-GST	RG	RG-GST
1.38 ± 0.22	4.33 ± 0.29 *	1.89 ± 0.09	2.40 ± 0.27	0.20 ± 0.05	0.25 ± 0.00

WR: white rice; BR: brown rice; RG: rice germ; GST: grain stabilization technique. Data are presented as means ± SEM (*n* = 3). Significant differences between non-processed and GST-processed samples are indicated by independent *t*-test (* *p* < 0.05).

**Table 3:** Changes in phytosterols (mg/100 g DW) and policosanols (mg/100 g DW) content in non-processed and GST-processed rice groups over a 90-day storage period.

Rice	Storage Duration	Phytosterols (mg/100 g DW)			Policosanols (mg/100 g DW)	
		Campesterol	Stigmasterol	ß-Sitosterol	Octacosanol	ɣ-Oryzanol
BR	Day-1	14.7 ± 1.12 ^a^	10.8 ± 0.58 ^a^	34.0 ± 2.72 ^a^	2.90 ± 0.22 ^a^	22.8 ± 0.18
	Day-30	11.8 ± 0.40 ^b^	8.19 ± 0.81 ^b^	28.6 ± 2.82 ^c^	2.33 ± 0.23 ^a^	23.3 ± 0.41
	Day-60	11.1 ± 0.17 ^b^	8.09 ± 0.54 ^b^	27.7 ± 1.22 ^c^	2.18 ± 0.10 ^b^	23.0 ± 0.60
	Day-90	9.2 ± 0.63 ^c^	8.74 ± 0.47 ^b^	30.1 ± 0.82 ^b^	1.83 ± 0.27 ^b^	22.0 ± 0.15
BR-GST	Day-1	17.3 ± 0.28 ^a^*	11.5 ± 0.82 ^a^	38.2 ± 1.94 ^a^	3.00 ± 0.23 ^a^	22.1 ± 0.64
	Day-30	15.8 ± 0.65 ^b^**	10.7 ± 0.89 ^a^*	36.5 ± 3.04 ^a^*	2.85 ± 0.18 ^ab^	23.0 ± 0.41
	Day-60	16.4 ± 0.57 ^b^***	10.8 ± 0.06 ^ab^*	37.1 ± 0.67 ^a^***	2.74 ± 0.28 ^b^*	21.4 ± 0.66
	Day-90	15.4 ± 0.79 ^c^**	10.1 ± 0.11 ^b^**	34.0 ± 0.79 ^b^**	2.63 ± 0.21 ^b^*	20.4 ± 0.82
WR	Day-1	3.81 ± 0.15	3.19 ± 0.15 ^b^	15.1 ± 1.65 ^ab^	0.44 ± 0.03	1.34 ± 0.03
	Day-30	3.42 ± 0.32	3.43 ± 0.13 ^a^	13.9 ± 0.22 ^a^	0.40 ± 0.05	1.34 ± 0.07
	Day-60	3.45 ± 0.06	3.24 ± 0.05 ^ab^	13.4 ± 0.36 ^ab^	0.39 ± 0.03	0.98 ± 0.03
	Day-90	3.20 ± 0.38	3.07 ± 0.15 ^b^	12.7 ± 0.72 ^b^	0.39 ± 0.02	1.06 ± 0.06
WR-GST	Day-1	3.48 ± 0.08 ^a^	3.58 ± 0.11 *	14.3 ± 0.31 *	0.36 ± 0.01 ^a^**	1.14 ± 0.06
	Day-30	3.26 ± 0.22 ^ab^	3.34 ± 0.06	13.4 ± 0.97	0.36 ± 0.01 ^a^	0.82 ± 0.03
	Day-60	3.07 ± 0.04 ^b^	3.58 ± 0.28	13.5 ± 0.71	0.34 ± 0.02 ^a^	0.76 ± 0.03
	Day-90	3.20 ± 0.15 ^b^	3.40 ± 0.09 *	13.1 ± 0.77	0.3 ± 0.03 ^b^*	0.91 ± 0.04
RG	Day-1	159.8 ± 9.03 ^b^	79.4 ± 3.62 ^a^	358.3 ± 26.6 ^a^	10.0 ± 0.35 ^a^	136.3 ± 2.40
	Day-30	160.4 ± 13.0 ^a^	78.1 ± 5.08 ^a^	359.3 ± 35.8 ^a^	9.39 ± 0.23 ^ab^	135.2 ± 0.25
	Day-60	158.8 ± 8.85 ^a^	76.4 ± 3.92 ^ab^	351.7 ± 22.4 ^ab^	9.00 ± 0.19 ^bc^	133.3 ± 3.10
	Day-90	143.1 ± 4.38 ^c^	71.0 ± 1.88 ^b^	318.6 ± 8.9 ^b^	8.43 ± 0.55 ^c^	125.9 ± 3.22
RG-GST	Day-1	178.9 ± 9.80 ^b^*	87.4 ± 4.16	391.6 ± 25.5 ^b^	10.2 ± 0.81	135.2 ± 3.74
	Day-30	200.0 ± 4.64 ^a^**	97.3 ± 3.01	435.6 ± 11.4 ^a^**	10.3 ± 0.31 *	138.2 ± 3.74
	Day-60	192.9 ± 8.74 ^a^*	90.9 ± 3.99	421.5 ± 14.8 ^ab^*	10.1 ± 0.62 **	133.3 ± 2.45
	Day-90	190.2 ± 2.14 ^ab^***	90.3 ± 1.11	412.6 ± 6.1 ^ab^***	10.1 ± 0.39 *	125.5 ± 0.39

WR: white rice; BR: brown rice; RG: rice germ; GST: grain stabilization technique. Data are presented as means ± SEM (*n* = 3). Different letters (a–c) indicate significant differences (*p* < 0.05) among time points within the same group. Significant differences between non-processed and GST-treated samples were verified by independent *t*-test (* *p* < 0.05; ** *p* < 0.01; *** *p* < 0.001).

**Table 4:** Bacterial counts (log CFU/g DW) and mycotoxins (µg/kg) in non-processed and GST-processed rice groups over 90 days of storage.

Storage Duration	Bacterial Counts (log CFU/g DW)				Mycotoxins (µg/kg)	
	Day-1	Day-30	Day-60	Day-90	Zearalenone	Ochratoxin A
BR	6.36 ± 0.06 ^a^	5.26 ± 0.02 ^b^	5.09 ± 0.00 ^c^	4.92 ± 0.01 ^d^	n.d.	n.d.
BR-GST	5.95 ± 0.04 ^a^**	5.10 ± 0.03 ^b^*	5.04 ± 0.00 ^b^**	4.71 ± 0.03 ^c^**	n.d.	n.d.
WR	3.39 ± 0.05 ^a^	2.83 ± 0.02 ^b^	2.15 ± 0.00 ^c^	1.62 ± 0.02 ^d^	n.d.	n.d.
WR-GST	2.73 ± 0.06 ^a^**	2.33 ± 0.03 ^b^**	1.36 ± 0.05 ^c^**	1.37 ± 0.05 ^c^**	n.d.	n.d.
RG	6.18 ± 0.04 ^a^	5.66 ± 0.05 ^b^	4.34 ± 0.00 ^c^	4.34 ± 0.00 ^c^	9.61	0.91
RG-GST	5.43 ± 0.06 ^a^***	4.33 ± 0.20 ^b^*	4.10 ± 0.01 ^b^***	3.56 ± 0.00 ^c^***	17.01	0.78

WR: white rice; BR: brown rice; RG: rice germ; GST: grain stabilization technique; n.d.: not detected. Data are presented as means ± SEM (*n* = 3). Different letters (a–d) indicate significant differences (*p* < 0.05) within non-processed and GST-processed groups. Significant differences between original grain and GST-treated samples were verified by independent *t*-test (* *p* < 0.05; ** *p* < 0.01; *** *p* < 0.001).

## Data Availability

The original contributions presented in this study are included in the article. Further inquiries can be directed to the corresponding author.

## Abbreviations

BR: brown rice, CFU: colony-forming unit, DON: deoxynivalenol, DW: dry weight, FAV: free acid value, FDA: American food and drug administration, GST: grain stabilization technique, MC: moisture content, MFDS: Ministry of Food and Drug Safety, OTA: ochratoxin A, RG: rice germ, RS: resistant starch, SEM: scanning electron microscopy, WR: white rice, ZEA: zeralenone.
